# Microbial Fabricated Nanosystems: Applications in Drug Delivery and Targeting

**DOI:** 10.3389/fchem.2021.617353

**Published:** 2021-04-20

**Authors:** Kumar Sachin, Santosh Kumar Karn

**Affiliations:** ^1^Department of Biosciences, Swami Rama Himalayan University, Dehradun, India; ^2^Department of Biochemistry and Biotechnology, Sardar Bhagwan Singh University, Dehradun, India

**Keywords:** nanomaterial, fabrication, drug delivery, biomineralization, microbes, nano-organics

## Abstract

The emergence of nanosystems for different biomedical and drug delivery applications has drawn the attention of researchers worldwide. The likeness of microorganisms including bacteria, yeast, algae, fungi, and even viruses toward metals is well-known. Higher tolerance to toxic metals has opened up new avenues of designing microbial fabricated nanomaterials. Their synthesis, characterization and applications in bioremediation, biomineralization, and as a chelating agent has been well-documented and reviewed. Further, these materials, due to their ability to get functionalized, can also be used as theranostics i.e., both therapeutic as well as diagnostic agents in a single unit. Current article attempts to focus particularly on the application of such microbially derived nanoformulations as a drug delivery and targeting agent. Besides metal-based nanoparticles, there is enough evidence wherein nanoparticles have been formulated using only the organic component of microorganisms. Enzymes, peptides, polysaccharides, polyhydroxyalkanoate (PHA), poly-(amino acids) are amongst the most used biomolecules for guiding crystal growth and as a capping/reducing agent in the fabrication of nanoparticles. This has promulgated the idea of complete green chemistry biosynthesis of nano-organics that are most sought after in terms of their biocompatibility and bioavailability.

## Introduction

Microorganisms have aptly been regarded as the most versatile living creatures on earth capable of executing any or all conceivable functions. Microbial cells generally need metal ions in micro-quantities as cofactors for proper functioning of metabolic enzymes and proteins. They, either, oxidize metals for deriving electrons and energy to sustain their living, or, reduce metal ions through metalloregulatory mechanism when subjected to presence of heavy metal contaminants that would otherwise become toxic to their survival (Park et al., 2016). Nanobiomedicine instituting application of microbially fabricated nanoparticles is an ever-evolving field in the nanosciences. The genesis of microbial fabrication of nanoparticles lies in the process of biomineralization. More than 60 elements have so far been recognized by various groups of microorganisms, converting them into complex nanoscale architecture much beyond the scope of any physical, chemical, and mechanical engineering means (Crichton, 2019). These biocomposites possess multifunctional properties highly desirable for biotechnological applications.

Metallic nanoformulations of microbial origin has been in focus lately due to their improved results and wide application in drug delivery (Delasoie and Zobi, 2019), biological tagging (Tang et al., 2019), biosensors, and pharmaceutical methods (Grasso et al., 2019). The inclination toward biogenic nanomaterial products is further augmented by its categorization under “green chemistry” synthesis methods that aims to minimize negative impact on the environment caused by use of toxic chemicals during the synthesis of metal nanoparticles by the conventional routes. Thus, the involvement of microorganisms including bacteria, fungi and viruses, plants and/or enzymes, proteins, polysaccharides obtained thereof, in obtaining nanoparticles of various elements becomes an ecologically friendly alternative and is currently an active area of research and development. Irrespectively, there are three basic ways in which synthesis of nanoparticles are approached: (i) dry nanotechnology - nanomaterials from carbon, silicon, and other inorganic materials are synthesized using physical chemistry methods; (ii) wet nanotechnology—in which biological systems are used for synthesis in liquid medium; and (iii) computational nanotechnology—process involving modeling and simulation study of nanomaterials (Le et al., 2016).

A number of reports have been documented discussing the process of synthesis of metal nanoparticles by microorganisms (Park et al., 2016; Salunke et al., 2016; Saratale et al., 2018) and their applications as nanocatalysts (Kratošová et al., 2019), sensoristic and biomedical field (Grasso et al., 2019). However, there is hardly any study on drug delivery aspect of biometallic nanoparticles. This article presents a focused review on the application of microbial fabricated nanosystems in biomedical field with a special attention to drug delivery and targeting.

## Biosynthesis of Metal Nanoparticles

The practice of biosynthesis of metal nanoparticles has been in vogue since the 1960's (Temple and Le Roux, 1964). Microorganisms belonging to different groups and genera including bacteria, actinomycetes, yeast, fungi, algae, microalgae, and viruses have been extensively employed for the synthesis of metal nanoparticles. Diversified microbes growing in extreme environment like *Deinococcus radiodurans* (Li J. et al., 2019), archaea (Srivastava et al., 2013) group as well as marine (Patil and Kim, 2018) ecosystem have been implicated in synthesis of metal nanoparticles. Nanoparticles of various metals, particularly those from heavy and toxic group, viz. Ag, Au, Cd, Se, Ni, Ti, Pd, Pt and metal oxides like Fe_3_O_4_, ZnO, Zirconia, TiO_2_, CeO_2_ along with their functional derivatives have been reported (Saratale et al., 2018).

The microgenic synthesis of nanoparticles involves simple and spontaneous biophysical and biochemical processes giving rise to stable and monodisperse formulations, though the exact *modus operandi* of the biosynthetic machinery at molecular level is still a gray area of research (Narayanan and Sakthivel, 2010). Microbes utilize mechanisms such as solubility changes, biosorption, bioaccumulation, extracellular precipitation, metal complexation, and chelation for the synthesis of metal nanoparticles along with involvement of reducing NAD(P)H-dependent enzymes viz. Nitrate reductase, Sulphite reductase, Cysteine desulfhydrase, and Glutathione (Ovais et al., 2018). Microorganisms are known to be rich in bioactive compounds that act as natural reductant while dealing with metal salts. Fundamentally, microbial biomineralization can occur by two processes. They are biologically induced by cell metabolic activity wherein the microorganisms do not possess much control over where and how the precipitate will form in the extracellular space. On the other hand, intracellular mineralization take place through a biologically controlled process in compartments within the cell and can be controlled in terms of size, texture, and orientation (Qin et al., 2020). Though, extracellular nucleation site of metal particles is a desirable trait citing ease of harvesting in case of large-scale production scenario. The nucleation site for biologically induced mechanism may be provided by the negatively charged exo-polysaccharide (EPS) secreted by microbes or some surface proteins that help in sequestering metal ions through ionic interactions. In case of intracellular biomineralization, the metal cation uptake take place through dedicated ion channels or pumps in membrane bound vesicles. The magnetite biomineralization in magnetotactic bacteria represents an excellent example of the intracellular process. Specific metal-binding proteins namely, metallothioneins and phytochelatins also assist in metal complexation (Cobbett and Goldsbrough, 2002).

The biogenic fabrication of metal nanoparticles using microbes is attached with several advantages, in terms of enzymatic reduction of metal ions into the zero-valent metals without any toxic chemical reagents and elaborate physical or chemical steps added with all processes carried out at ambient temperature. The only requirement, it seems, is the maintenance of a sterilized environment and culture conditions. The culture conditions must be optimal for light, temperature, pH, and growth nutrients and a sterilized environment should be maintained to prevent cross-contamination by other microorganisms.

**Figure 1:**
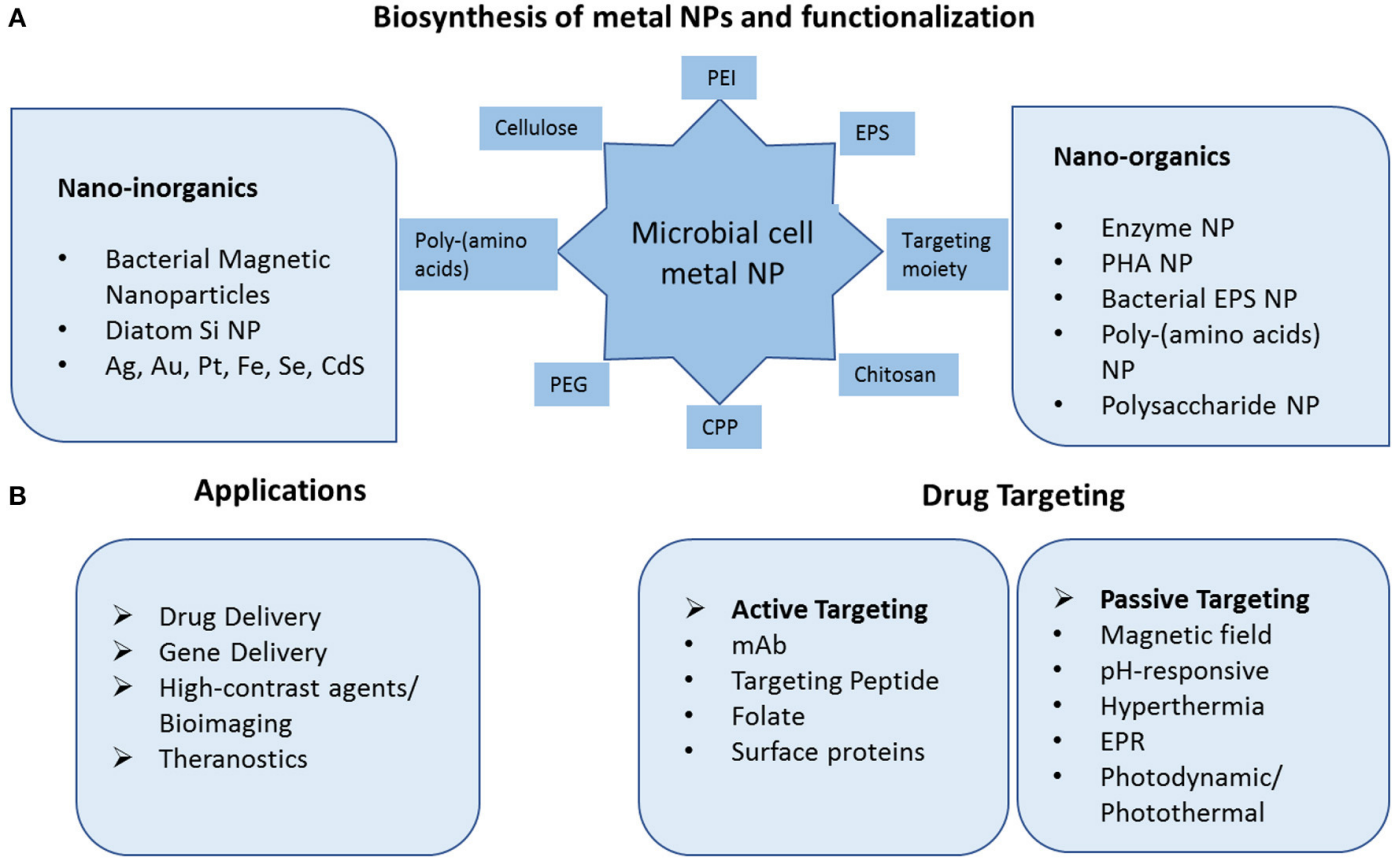
Schematic diagram of microbial fabricated nanosystems and their application in drug delivery and targeting. **(A)** Biosynthesis of inorganic metal nanoparticles and biopolymeric nanoparticles. The nanoparticles can be functionalized with variety of peptides, polynucleotides, amino acids, targeting moiety. **(B)** Application of nanoparticles in drug and gene delivery via active and passive mode of targeting and in high quality diagnostic techniques as contrast agents. PEI Polyethyleneimine, EPS ExoPolySaccharides, CPP Cell Penetrating Peptide, PEG Polyethylene Glycol, PHA PolyHydroxyAlkanoates, mAb Monoclonal Antibody, EPR Enhanced Permeability, and Retention.

With an aim to understand molecular details of Gold nanoparticle synthesis by the bacterium *Lactobacillus casei* Kikuchi et al. (2016), demonstrated the role of glycolipids in reducing Au(III) to Au(0) and in synthesizing nanoparticles of smaller size. Such work could be helpful in devising new ways in which Gold nanoparticles could be synthesized within microorganisms. The size of metal nanoparticles can be regulated by factors such as concentration of the precursor, the pH, the temperature, and the reaction kinetics (Poulose et al., 2014). The stability may be attributed to use of biopolymers as capping/reducing agents and water as solvent. Popular biomolecules such as amino acids, peptides, proteins, polysaccharides, lipids, nucleic acids, and citric acid are amongst the most used for channelizing crystal growth and also as a capping agent in the fabrication of nanoparticles (Sable et al., 2020). A comparative analysis of the different capping layer composition was carried out with SeNP of five bacterial strains and hypothesized differential binding capabilities of the capping biomolecules to the core metalloid matrix (Bulgarini et al., 2020). A schematic representation of a microbial cell capable of forming functionalized metal nanoparticles and their biomedical applications is illustrated in Figure 1.

Although bacteria have cornered more attention in metal biomineralization, contributions from fungal (Hu et al., 2019) and algal (AlNadhari et al., 2020) species cannot be ignored. Fungi is known to secrete many enzymes and organic acids, particularly oxalic acid, that play a pivotal role in metal biotransformation forming metal complexes such as metal-oxalates. The general mechanism involves metal reduction by redox mediators followed by stabilization by capping agents to prevent aggregation. Yeasts are rarely involved in natural mineralization process. Nevertheless, they exhibit tolerance to adverse culture conditions including metal toxicity. They also have the ability to distinguish between metals such as Mercury, Antimony, and Selenium depending on their toxicity (Gadd and Pan, 2016). The mechanism of biomineralization of metal nanoparticles by bacteria, magnetotactic bacteria, fungi, yeast, diatoms has been elaborately described elsewhere (Qin et al., 2020).

## Applications in Drug Delivery and Targeting

Though metal nanoparticles of microbial origin have found wide ranging applications, not many have been applied for drug delivery and targeting purposes. Novel drug delivery systems basically employ nanocarriers to improve pharmacokinetics and/or pharmacodynamics, bioavailability of the conventional drugs or modern therapeutic agents. This is particularly necessary for cancer therapy. The tumor physiology presents typical microenvironment that prevents and resists optimal diffusion of chemotherapeutic drugs due to high interstitial fluid pressure. At the same time, passive delivery of drugs is facilitated due to Enhanced Permeability and Retention Effect (EPR) exerted by the leaky tumor vasculature as well as faulty lymphatic drainage. Hence, different external energy sources such as sound, light (photodynamic therapy), magnetic field, temperature (hyperthermia), pH-sensitive in solo or in combination are intensively being explored. Further, tumors develop hypoxic regions due to their high rate of cell division and metabolism.

For nanoparticles to be applicable in drug delivery, desirable characteristics like biocompatibility, non-toxicity, drug/gene entrapment efficiency, and scope of getting functionalized with biomacromolecules such as peptides, proteins, lipids, and targeting moiety, must be present (Wuttke et al., 2017). A summary of the different nanosystems synthesized by microorganisms and their application has been summarized in Table 1.

**Table 1:** Summary of microbial fabricated nanomaterials for drug delivery systems.

**Organism**	**Metal NP**	**Type of formulation**	**Application**	**References**
*Magnetospirillum gryphiswaldense*	-	BM-Ab complex	Active targeting against tumor mice model	Tang et al., 2019
Magnetotactic bacteria	Bacterial magnetic nanoparticle (BMP)	BMP-PEI/DNA	Gene delivery	Xiang et al., 2007
Magnetotactic bacteria	Bacterial magnetosome	Drug-loaded magnetosome	Anticancer drug delivery	Geng et al., 2019
Magnetotactic bacteria	Bacterial magnetosome	Genipin (GP) and poly-l-glutamic acid (PLGA)-modified bacterial magnetosome	Anticancer drug delivery	Long et al., 2016
Magnetotactic bacteria	Bacterial magnetosome	BM-PEI-siRNA	Anticancer Gene delivery	Dai et al., 2017
Magnetotactic bacteria	Bacterial magnetosome	Plasmid/Drug loaded BM	Drug/Gene delivery	Cheng et al., 2016
Magnetotactic bacteria	Au nanorods	BMP-Au rods-folic acid	Theranostic agents	Nima et al., 2019
*Magnetospirillum magneticum* AMB-1	Bacterial magnetosome	Protein functionalized BM	Labeling tumor markers	Kotelnikova et al., 2018
Diatom	Modified Diatomite NP	Dual-biofunctionalized, PEG and Cell Penetrating Peptide	Delivery of anticancer drug, Sorafenib	Terracciano et al., 2015
*Magnetococcus marinus* strain MC-1	-	Drug-loaded nanoliposome	Delivery of multiple drug agents	Felfoul et al., 2016)
Diatom	Diatomite NP	siRNA bioconjugated	siRNA delivery, gene silencing	Rhea et al., 2014
Diatom	Modified Diatomite NP (DNP)	Idiotype-specific peptide-DNP	Active targeting of siRNA; lymphoma	Martucci et al., 2016
Diatom	Iron Oxide	Iron Oxide NP encapsulated diatom vehicle	Passive drug delivery under magnetic field	Todd et al., 2014
Diatom	Graphene Oxide-Diatomaceus earth	Nano-hybrid	pH sensitive drug delivery	Kumeria et al., 2013
Diatom	Au NP	PEG-Diatom-AuNP nanocomplex	Imaging nanodevice	Terracciano et al., 2018
Diatom+Bacterial magnetic nanowire	SiNP	Hybrid microsphere	Dual drug chemotherapy	Maher et al., 2017
Diatomaceous earth microparticle	-	DEMP-β-CD:Ad	pH-responsive drug delivery	Kabir et al., 2020
*Lactobacillus plantarum*	AuNP	Antibiotic-AuNP-EPS	Drug delivery against MDR	Pradeepa et al., 2016
*Aspergillus niger*	-	Glucose oxidase-NP	Biosensor; Serum glucose determination	Kundu et al., 2013
*Candida sp*.	-	Recombinant Uricase-NP	Biosensor; uric acid	Chauhan et al., 2014
Microbial PHA	-	P(3HB-3HHx) NP-PhaP-EGFR targeting peptide	Active targeting	Fan et al., 2018
Yeast cells		nHAP-yeast with folic acid	Active targeting	Ma et al., 2018
Microbial PHA	-	P(3HB-3HHx) NP	Drug delivery; 5-FU	Lu et al., 2010
*Lactobacillus plantarum*	AuNP	Bacterial EPS stabilized NP	Drug delivery; antibiotic	Pradeepa et al., 2016
*Halomonas maura*	-	Chitosan-Mauran EPS nanocomposite	Drug delivery; 5-FU	Raveendran et al., 2013
*Gluconacetobacter liquefaciens* (probiotic bacteria)	AuNP	GNP-CK-CopA3	Active targeting	Liu et al., 2020
Bacterial EPS	-	Gellan gum based floating bead	Drug delivery; antibiotic	Rajinikanth and Mishra, 2009
Bacterial EPS	Magnetic NP	MNP-Gellan gum/Mauran nanocomplex	Drug delivery and targeting	Sivakumar et al., 2014
Microbial poly-(amino acids)	-	Poly(γ-glutamic acid) (PGGA) NP	Drug delivery; antibiotic	Portilla-Arias et al., 2009
*Oscillatoria limnetica*	Ag-NPs	-	Biiomedical	Hamouda et al., 2019
*Ustilago maydis*	AuNPs	Mannosylerythritol lipid (MEL)	Biomedical	Bakur et al., 2019

### Bacterial Magnetic Nanoparticle

Superparamagnetic iron-oxide nanoparticles are popularly engaged in designing magnetically controlled drug delivery systems, biosensors and as contrast agents in MRI diagnosis (Ashraf et al., 2019). Magnetosomes, a unique lipid bound intracellular organelles found exclusively in magnetotactic bacteria, exhibit some special characteristics that find utmost favor among drug delivery researchers. Features such as regular morphology, narrow size distribution, low toxicity profile, resistance to agglomeration, makes them excellent carriers for drug / gene delivery applications. These nano-meter sized crystals are naturally synthesized through the process of cytoplasmic membrane invagination followed by influx of certain proteins and iron, leading to biomineralization of magnetite crystals (Firlar et al., 2019).

**Figure 2:**
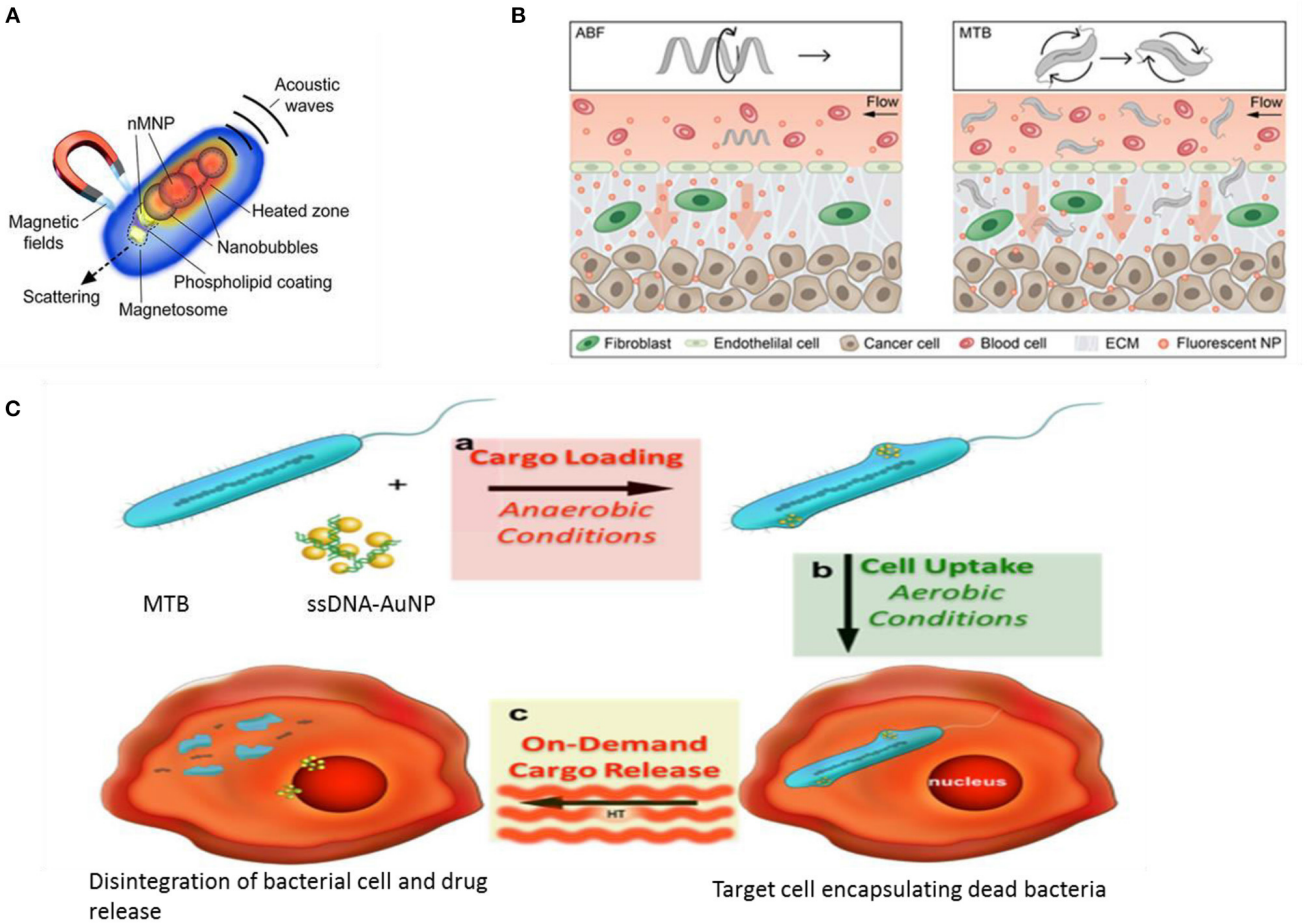
Magnetotactic bacteria (MTB) and drug delivery applications. **(A)** MTB as a multimodal photoacoustic, photothermal, and photomechanical contrast agent for single cell diagnostic and therapy (Nima et al., 2019). **(B)** Conceptual diagram depicting a single microrobot, the Artificial Bacterial Flagellum ABF, enhancing mass transport of NPs at the vessel -tissue interface (left), and swarms of MTB generating convective flow to improve mass transport (right). ECM ExtraCellularMatrix (Schuerle et al., 2019). **(C)** Drug delivery using physiological limitation of MTB under hyperthermia radiation (Alsaiari et al., 2016).

Magnetotactic bacteria are mostly Gram-negative belonging to the α-Proteobacteria group, with anaerobic or micro-aerobic type of metabolism (Vargas et al., 2018). They are natural producers of iron oxide (magnetite) and iron sulfide (greigite) nanoparticles bound by a lipid bilayer. The magnetosomes inside which these particles are harbored, primarily work to align the cells along external magnetic fields and guide the cells toward optimal nutrient or oxygen conditions. These particles can be isolated from bacterial cells and have proved useful in medical applications like peptide screening in drug development (Tanaka et al., 2008), anticancer drug delivery (Long et al., 2016; Geng et al., 2019), and gene therapy (Dai et al., 2017). These specialized bacteria have become popular bioresources as smart drug delivery systems against cancer patients (Kuzajewska et al., 2020). They are most preferable as passive drug delivery agents where drug or a gene fragment can be directed toward affected tissue under the influence of magnetic energy and hyperthermia. They can be easily combined with other heavy metal NPs such as Gold or functionalized with different ligands such as enzymes, proteins, nucleic acids, green-fluorescent protein, antibodies, and drugs to improve its range of applications. Experiments with bacterial strain *Magnetospirillum gryphiswaldense* have demonstrated that the chain alignment of the magnetosome seems perfect to enhance the hyperthermia effect in cancer therapeutics (Gandia et al., 2019). A study conducted on animal cancer model showed enhanced efficacy as MRI-contrast agents for the magnetosomes in comparison to Iron Oxide nanoparticles (Erdal et al., 2018). In a smart chemotherapy approach, a new heat sensitive system based on bacterial magnetosome was developed. A recombinant heat shock protein plasmid was constructed along with the anticancer drug Doxorubicin, which under hyperthermia treatment, brought about significant inhibition of osteosarcoma growth (Cheng et al., 2016). This construct was enabled only due to ionic interaction between the negatively charged phospholipid on the magnetosome membrane and positively charged plasmid and the anticancer drug. The magneto-aerotactic behavior of the MTB, *Magnetococcus marinus* strain MC-1 was exploited to deliver as many as 70 drug-loaded nanoliposomes to such Oxygen-depleted regions which are difficult to access. Upto 55% of the drug-loaded cells could be demonstrated to penetrate the colorectal xenograft in SCID-mice (Felfoul et al., 2016). Bacterial magnetic nanoparticles (BMP) in the size range 45–55 nm coated with polyethyleneimine (PEI) was effectively used to transfect DNA in mammalian cell lines as well as *in vivo* with reduced cytotoxicity and high expression efficiency (Xiang et al., 2007). Active targeting agents were also formulated in a recent study; bacterial magnetosome was complexed with an anti-tumor antibody (BM-Ab) resulting in greater tumor protection under magnetic therapy than did other methods of treatment (Tang et al., 2019). Magnetic and Au nanoparticles have been devised as theranostic devices for application in imaging and drug delivery (Rizzo et al., 2013). Nanohybrids of particles derived from magnetotactic bacteria with gold nanorods and folic acid served as effective theranostic agents enabling detection and photomechanical killing of breast cancer cells (Nima et al., 2019). These natural nanoparticles were applied as high-contrast probes much sought after in single-cell diagnostics as well as photothermal agents for single-cell therapy (Figure 2).

In an advanced engineering approach, artificial bacterial flagellum and live magnetotactic bacteria *Magnetococcus maneticum* strain AMB-1 was directed to inaccessible tumor tissues by exploiting the phenomena of ferrohydrodynamics. The two distinct approaches combined to act as micropropellers enhancing the co-delivery of NPs resulting in increased extravasation and tissue penetration (Figure 2) (Schuerle et al., 2019).

Recent molecular studies have highlighted a number of genes and proteins that play important roles in bacterial magnetic particles' synthesis and biomineralization. Further, genetic engineering techniques have enabled creation of fusion proteins, in which certain foreign protein domains could be anchored onto the natural transmembrane proteins associated with the magnetosome like Mms13, Mms16, and MamC and then applied for targeting or biomarker recognition agents (Yoshino et al., 2010). Thus, instead of chemical modifications on the magnetosome that might exert toxic effects, genetic engineering modifications allow expression of functional proteins on the magnetosome membrane. In a recent experiment, a biomodified magnetite nanoparticle was developed using the Mms6 protein and the barnase^*^barstar high-affinity protein pair to efficiently recognize and target HER2/neu tumor marker on the surface of cancer cells (Kotelnikova et al., 2018). In another interesting experiment, unmodified magnetotactic bacteria was formulated as DNA carriers using AuNP as a transmembrane facilitator to endocytosis. The gene fragment was loaded under anaerobic condition when the bacteria remains alive and then is exposed to air (killing the cells) where magnetic hyperthermia causes rupture of the cells releasing the encapsulated DNA (Figure 2) (Alsaiari et al., 2016).

### Diatoms Si Nanosystems

No study on microbial fabrication of nanoparticles can be complete without special focus on diatoms. Diatoms are unique photosynthetic unicellular microalgae that provide a natural source of mesoporous silica which turn out to be good candidates for drug delivery applications (Uthappa et al., 2018; Delasoie and Zobi, 2019). The production of biogenic silica begins with internalization of the soluble form of silicon, orthosilicic acid Si (OH)_4_ using specific transporter proteins. Inside specialized intracellular compartments, named silica deposition vesicles (SDVs), Si (OH)_4_ is converted into SiO_2_ network with the help of long chain polyamines, silaffin proteins, and silacidin peptides (Lechner and Becker, 2015). The diatoms' cell wall, collectively known as the “frustule” has been one of the most impressive examples of natural 3-D architecture of highly differentiated amorphous silica (SiO_2_) (Grachev et al., 2008). The siliceous frustule makes up the diatom skeleton which comprises the nanoscaled hypotheca and epitheca valves; it is this special structure that assigns it an attractive template for drug delivery applications. Moreover, reactive silanol (SiOH) groups can chemically modify the frustule surface, thus improving drug loading/release kinetics. Addition of other reactive groups (-NH_2_, -COOH, -SH, and -CHO) can further be utilized in several biomolecule (e.g., enzymes, proteins, antibodies, peptides, DNA, aptamers) conjugation (Rhea et al., 2017).

**Figure 3:**
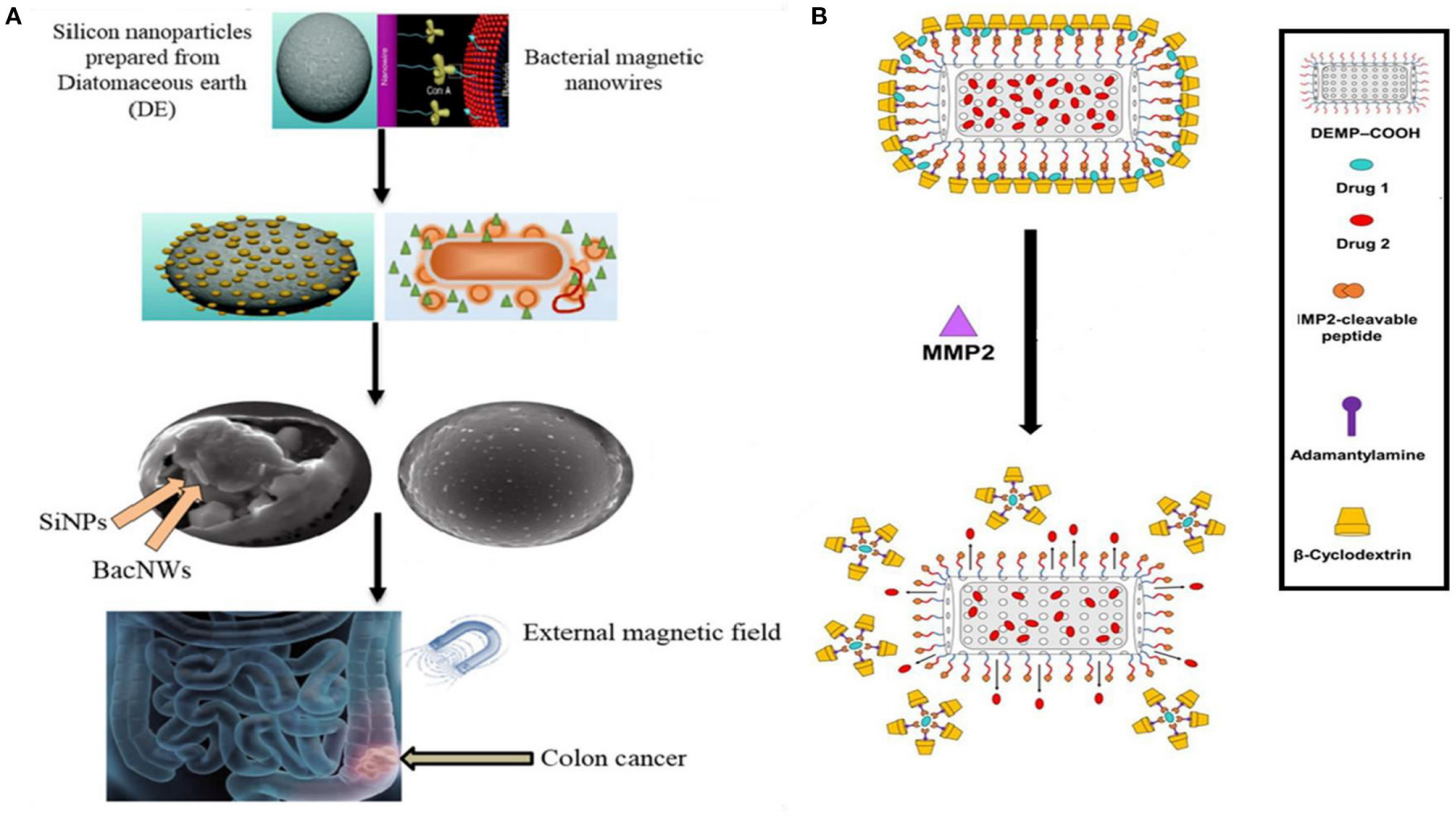
Application of Diatom based NPs in drug delivery. **(A)** Dual drug chemotherapy approach using Diatomaceous SiNP and bacterial magnetic nanowires. The drugs are released specifically in the colon region in pH responsive manner and under magnetic field (Maher et al., 2017). **(B)** The drugs were encapsulated in Diatomaceous Earth Microparticles (DEMP) either in siliceous core or in β-CD and Adamantane (β-CD:Ad) micelles. Matrix MetalloProteinases-2 (MMP-2) specific peptide substrate was used as a linker between DEMPs core and βCD:Ad shell for stimuli responsive drug release into tumor tissue (Kabir et al., 2020).

Terracciano (Terracciano et al., 2015) reported a study on diatomite nanoparticles (DNPs) based on PEGylation and peptide bioconjugation so as to enable solubilization of the otherwise insoluble anticancer drug Sorafenib, thereby achieving enhanced cellular uptake, loading/release kinetics. A dual drug chemotherapy is often desired in treatment of resistant tumors; however, the desired outcome, synergistic effect of the drugs in combination becomes a challenge. A diatomaceous earth embedded gel system was developed encapsulating the two drugs in different compartments thus controlling the release rate at optimal molar ratios *in vitro* (Figure 3) (Kabir et al., 2020). In another classical experiment achieving successful dual drug chemotherapy against colorectal cancer, anticancer agents 5-FU and Curcumin were loaded onto diatom-derived porous SiNP and magnetic bacterial nanowires. The two systems were then combined together using droplet-based microfluidics. The targeted release of the drugs was further ensured by fabricating the microspheres using hypromellose acetate succinate polymers, which are insoluble in the acidic medium of the stomach but soluble at basic pH (colon and rectum) (Figure 3) (Maher et al., 2017).

The first successful attempt at siRNA delivery with effective gene silencing using DNPs was demonstrated by Rhea et al. (2014). In a similar context, a new customized B-cell lymphoma therapy based on target-specific receptor-mediated DNPs was reported by Martucci et al. (2016) as gene delivery systems.

The design of multifunctional platforms integrating multiple components at nano scale to create hybrid nanodevices have also been attempted with diatoms. Todd et al. (2014) reported, for the very first time, Iron oxide nanoparticles encapsulated within the surface of diatom frustules for *in vivo* delivery of anticancer molecules as a magnetically active device. The results demonstrated sufficient accumulation of the drug at tumor site, almost six times higher than the control, under the influence of magnetic field. Losic et al. (2010) fabricated magnetically guided drug carriers for dopamine delivery using electrostatic interaction between the cationic Fe_3_O_4_ magnetic complex and anionic diatom frustules. Smart hybrid diatom-based devices incorporating diatomaceous biosilica with inorganic materials like titanium dioxide, semiconducting (Si-Ge), and metal (Au) scaffolds have been reported (Maher et al., 2018; Terracciano et al., 2018). A hybrid Au-diatomite NPs as innovative multifunctional device for imaging and drug delivery purposes was obtained (Terracciano et al., 2018). The nanostructure exhibited favorable optical properties, giving a clear surface plasmon resonance band in the UV-vis spectra at 550 nm.

Diatoms have also been studied as an exceptional biotemplate for drug delivery applications owing to species-specific ordered architecture and flexibility for surface modifications (Chao et al., 2014). Some of the best illustrations of biotemplating are formation of metal nanowires due to intercalation of metal complexes in DNA strands (Li N. et al., 2019), Tobacco Mosaic Virus as the viral template to fabricate nanocrystals (Wnek et al., 2013) and replicating morphology of pollen grains to create TiO_2_ microspheres (Yang et al., 2014). It is high time that the full potential of diatoms based drug delivery systems (DDS) is unfolded by necessary authorization in biomedicine application just as amorphous silica from diatoms have been approved for use in food and agriculture industry by the US FDA and is Generally Recognized as Safe (GRAS, 21 CFR Section 573.340).

### Other Microorganisms Nanoparticles

**Figure 4:**
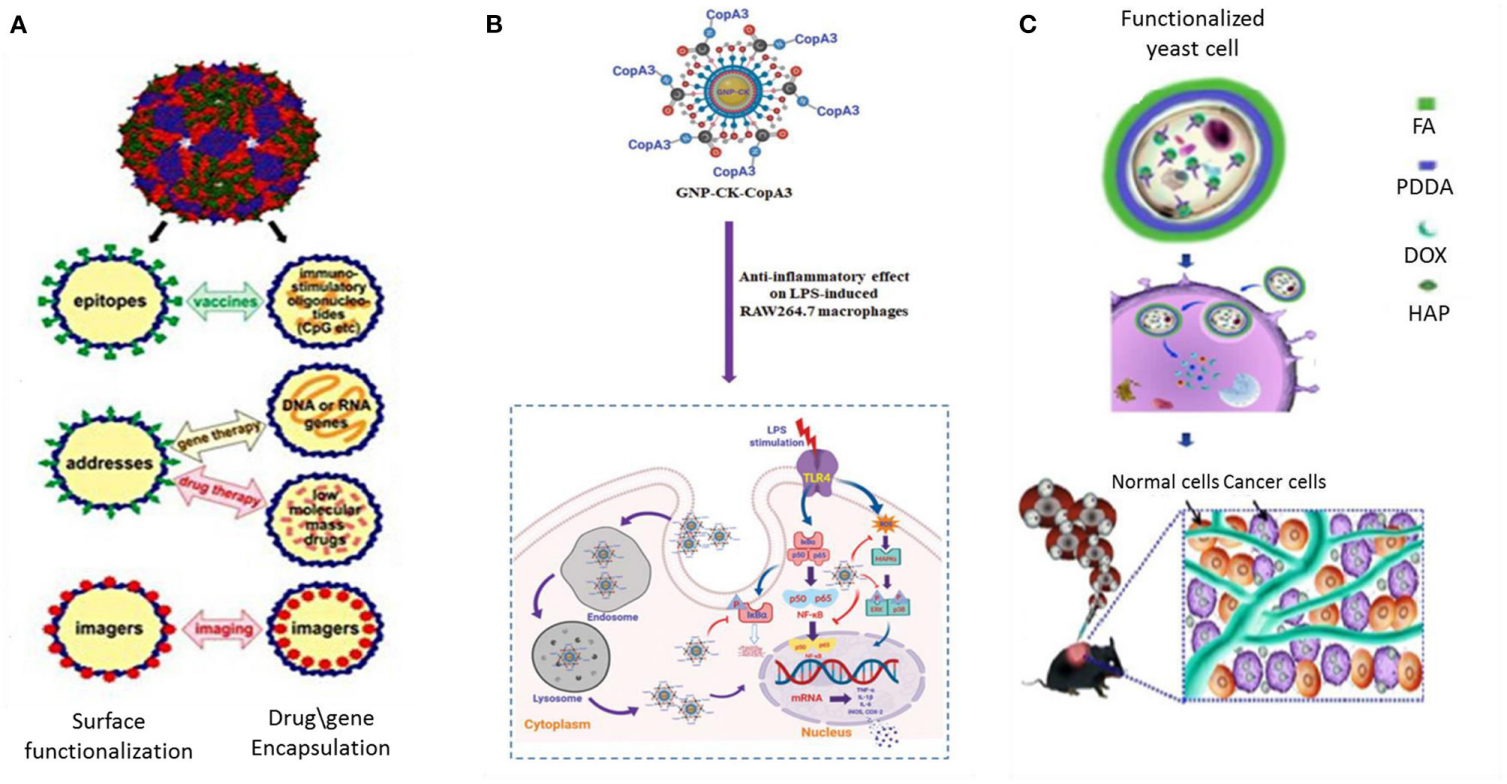
Application of viruses, bacteria, and yeast cells in drug delivery and targeting. **(A)** RNA phage VLP as scaffold for foreign epitopes or encapsulating immunostimulating adjuvants (CpG) in vaccine delivery, drug or gene delivery and as bioimaging agents for MRI and PET based diagnosis (Pumpens et al., 2016). **(B)** Active targeting of a biosynthesized peptide CopA3 surface conjugated and ginsenoside compound K (CK) loaded gold nanoparticles (GNP-CK-CopA3). The particles, once internalized in macrophages, efficiently suppressed expression of pro-inflammatory cytokines through inhibition of NF-κB and MAPK signaling pathways (Liu et al., 2020). **(C)** Biosynthesis of HydroxyAPatite (HAP) nanoscaffold inside yeast cell for dual responsive drug delivery against tumor (Ma et al., 2018). VLP Virus Like Particles, FA Folic Acid, PDDA Poly- (Diallyl Dimethyl Ammonium chloride).

Although magnetotactic bacteria and diatoms have drawn privileged focus in terms of designing numerous drug delivery applications, several other microorganisms including bacteria, yeast, fungi, and viruses have also been reportedly used in drug delivery formulations. There are numerous reports of biosynthesis of Gold and silver nanoparticles (El-Sherbiny and Sedki, 2019; Huang et al., 2019; Liu et al., 2020). AuNPs are applied due to their unique opto-electronic properties as well as low toxicity; while AgNPs are well-known for their antimicrobial activity. However, it is the antiangiogenic property of these metal NPs that is of relevance in potential biomedical applications. In order to study anti-angiogenic property of metal nanoparticles, Ag, Au, and Si as well as C nanostructures were assessed, among which Au NPs of avg size 20 nm and spherical shaped particles were found to cause maximum effect on the tumor cells (Saeed et al., 2019). AuNP and AgNP produced by the reduction of gold chloride (AuCl_4_) in *Stenotrophomonas maltophilia* (Huang et al., 2019) and reduction of silver nitrate (AgNO_3_) using optimized nitrate reductase fermentation in *Bacillus licheniformis* (Vaidyanathan et al., 2010) respectively, show good antitumor potential by way of inhibiting VEGF-mediated angiogenesis (Kalishwaralal et al., 2009). In particular gold particles work best in the photothermal destruction of tumor cells (Dhanalekshmi et al., 2020). Probiotic bacteria are known to be beneficial for human health. Researchers took the services of these biofriendly bacteria to synthesize a hybrid peptide-AuNP loaded with ginsenoside compound K (CK) (GNP-CK-CopA3). The intracellularly placed nanoconjugate was used to specifically target the macrophages and brought considerable reduction in Reactive Oxygen Species (ROS) and pro-inflammatory cytokines at genetic level (Liu et al., 2020) (Figure 4).

Among the metal nanoparticles, Silver AgNPs have gained paramount interest due to their unique physicochemical and biological characteristics. Moreover, advancement in synthetic chemistry have led to development of many peptide-based therapeutics in the market (Kaspar and Reichert, 2013). The two concepts when combined together form “value-added” constructs with new important properties like increased potency, site of action targeting capability, decreased toxicity not previously possessed by the NPs alone. Some commonly applied peptides conjugated to AgNPs are transactivator of transcription (TAT) and TAT-like peptides, Arginylglycylaspartic acid (RGD) peptides, the pep-1 peptide. The peptide conjugated NPs have found numerous applications in biomedical field including intracellular delivery, drug delivery, and cancer therapy (Ramesh et al., 2016). In another study, kappa-selenocarrageenan, the organic Selenium, served as reducing and capping agent for biosynthesis of a multifunctional AgNP that might have great applications in multiple areas like medicine, disease diagnostic, and drug delivery (Jin et al., 2019). An aqueous extract of the cyanobacteria *Oscillatoria limnetica* fresh biomass was used for the green synthesis of Ag-NPs. It exhibited strong antibacterial activity against multidrug-resistant bacteria (*Escherichia coli* and *Bacillus cereus*) as well as cytotoxic effects against both human breast (MCF-7) cell line and human colon cancer (HCT-116) cell line (Hamouda et al., 2019).

Selenium nanoparticles (SeNPs) confer low cytotoxicity compared to Se compounds and possess excellent anticancer and therapeutic properties making them appropriate for different medicinal applications (Wadhwani et al., 2016). Biogenically made SeNPs remain stable due to natural organic coating and do not aggregate with time. These nanoparticles may be localized extracellular, intracellular, or membrane bound depending on the source and culture conditions. Cellular uptake and anticancer activity were evaluated of surface derivatized Spirulina polysaccharide SeNPs that inhibited cell growth through induction of apoptosis, DNA fragmentation and chromatin condensation (Yang et al., 2012).

Green synthesis of Platinum (Pt) NPs using herbal extracts, brown seaweed *Padina gymnospora*, Gram positive *Streptomyces* bacterial strains as well as fungal *Fusarium oxysporum* were assessed. The metallic NPs from the bacterial and algal species exhibited significant cytotoxicity when tested against human breast cancer cell line MCF-7 and A549 lung carcinoma cell lines respectively. They were also found compatible with red blood cells with no observed hemolysis implying their potential biomedical applications (Fahmy et al., 2020). Cadmium sulfide (CdS) nanocrystals were synthesized by the non-sulfur bacterium *Rhodopseudomonas palustris* growing on cadmium sulfate (CdSO_4_) (Bai et al., 2009) which were used in the design of biosensing devices for molecular diagnosis (Daneshpour et al., 2018). A cell-free supernatant of actinobacteria was used to synthesize Fe-NP revealing its strong antioxidant and antimicrobial activity. The results on its cytotoxic effect on prostate cancer cell lines illustrated its utility in a wide range of biomedical field (Rajeswaran et al., 2020). In a yet to be published article, iron-oxide nanoparticle biosynthesized by a unique halophilic archaea Halobiforma sp. N1 has been reported. The product is being optimized for large scale production as a potential hyperthermia treatment for cancer therapy (Salem et al., 2021).

Alike Diatoms and viruses, yeast cells could also be used as a biotemplate core to synthesize Strontium loaded microspheres. It had good biocompatibility and served as a potential drug release system for the bone regeneration field (Huang et al., 2016). As a biotemplate, yeasts were used to fabricate another biocompatible Mg-doped CaCO_3_ microcapsules (Li et al., 2017). Due to higher biosorption capacity toward metallic ions the composite microcapsule revealed better cytocompatibility when co-cultured with mouse mesenchymal stem cells. An advanced and intelligent yeast cell based intracellularly fabricated hydroxyapatite nanoscaffold carrier was constructed with a large loading capacity for drugs (Figure 4). The nHAP-yeast was further functionalized with folic acid showed dual responsive release profile as the anaerobic microorganism tends to seek oxygen deficient environment prevailing in tumor space and folate-dependency character of tumor drives the particles to tumor site (Ma et al., 2018).

The unique capability of viral proteins to assemble as supramolecular cages and the possibility of displaying specific molecular ligands can lead to production of highly organized, functionalized nano-vehicles for drug delivery (Petrenko and Jayanna, 2014) (Figure 4). Some of the characteristics that phages possess such as the monodispersity of phage particles, their production in easily cultivable bacterial species, their self-assembling feature and the different morphological types of particles, make them interesting nanoparticulate entities, although phages themselves have not been explored as biomaterials. Viruses tend to adopt self-mineralization under metal-ion-abundant conditions as a means of bioprotection while they are off their host cells (Wang et al., 2018). Much attention has been given to virus-mediated biosynthesis of nanoparticles *via* biomimetics. Bacteriophages including Tobacco Mosaic Virus (TMV) (Wnek et al., 2013) and other viruses can be easily engineered into different nanoconstructs. SiO_2_ nanocapsules were synthesized using CowPea Mosaic Virus (CPMV) as template at room temperature without using any catalyst or surfactant (Kumar et al., 2015). The concept of using RNA phages as encapsulating and targeting capsid agents was developed as early as in the 1990's. RNA phage virus-like particles demonstrating coupling of coat protein with different organic (peptides, oligonucleotides, and carbohydrates) and inorganic (metal ions) compounds have been applied as nanosystems for targeted drug delivery, and bioimaging tools (Pumpens et al., 2016). In addition, metal-based nanowire constructs with phages as scaffolds have long been fabricated *in vitro* with potential applications in different biomedical fields, including biosensing (Harada et al., 2018).

### Nano-Organics

The diversity of microbial metabolic pathways has opened up an enthusiastic opportunity to exploit microbial cells as “biofactories” to produce nanostructured and functional agents (Choi et al., 2018). Microbial population can well be utilized for their ability to chelate and assemble metals into organized crystalline nanostructures as discussed in previous sections; alternately, the cells can become sources of metabolites like biopolymers that can be biofabricated as nano-organic materials with appealing applications in biomedicines. These biopolymers are mostly biologically favored polymers that would get internalized in mammalian cells and rapidly degraded asserting their suitability as clinical applicants.

Microbial enzymes are known to have wide industrial application ranging from textile, leather, food-based industries, and paper and pulp industry, cosmetic and detergent industry. Immobilization of enzymes onto both organic and inorganic supports achieves greater productivity, capability of automation, and scale up. These microbially-derived catalytic molecules can also be aggregated into clustered assembly of protein structure in nanometer dimension. Such Enzyme Nanoparticles (ENPs) exhibit unique physicochemical properties, besides, increased surface area for catalytic activity (Yadav et al., 2018). The enzyme nanoparticles have been prepared using simple desolvation techniques akin to biopolymeric gelatin nanoparticle synthesis (Hathout and Metwally, 2019) followed by crosslinking within their self in a controlled fashion. These nanoparticle aggregates can be functionalized and then immobilized bringing about leap fold improvement in their overall application process. Direct immobilization of enzyme nanoparticles has been well-applied as a promising strategy for designing highly efficient biosensing or biomedical devices, enzyme reactors and biofuel cells. Liu et al. (2005) reported for the first time the covalent immobilization of thiolated horseradish peroxidase NPs aggregated onto Au electrode. Thereafter, several microbial enzymes formulated into nanoparticle aggregates and employed in making biosensors such as glucose oxidase from *Aspergillus niger* (Kundu et al., 2013), cholesterol oxidase (Aggarwal et al., 2016) and uricase from *Candida sp*. expressed in *Escherichia coli* (Chauhan et al., 2014). The preparation, characterization and application of enzyme nanoparticles have been well-documented by Prof Pundir (Yadav et al., 2018).

Polyhydroxyalkanoates (PHA) constitute another microbial derived polymer attractive for biomedical applications due to their exceptional biodegradability and biocompatibility (Li and Loh, 2015). Both Gram-positive and Gram-negative bacterial genera such as *Pseudomonas, Bacillus, Ralstonia, Aeromonas, Rhodobacter*, among others, can be sourced for obtaining PHA (Rehm and Steinbüchel, 1999). Microbes generally hold PHAs as insoluble granules in inclusion bodies as a carbon and energy source. Great biocompatibility, non-cytotoxicity and its aliphatic nature makes them a prospective polymeric nanocarrier for controlled drug release of difficult to administer hydrophobic compounds (Li and Loh, 2015). Some bacterial PHAs, such as poly-3-hydroxybutyrate P(3HB), 3-hydroxybutyrate and 3-hydroxyvalerate co-polymers P(3HB-3HV), poly-3-hydroxyoctanoate P(3HO), and poly-3-hydroxybutyrate-co-3-hydroxyhexanoate P(3HB-3HHx) are useful in tissue engineering, in wound dressing, and as carriers, scaffolds and drug delivery platforms (Singh et al., 2019). Lu and his team fabricated an emulsifier free P(3HB-3HHx) based nanoparticles using a modified solvent diffusion method (Fan et al., 2018). Emulsifiers are known to be difficult to eliminate even after repeated washing during preparation of polymeric NPs (Shakesheff et al., 1997). This P(3HB-3HHx) NP was wholly biofunctionalized using the PHA granule-associated protein PhaP fused to an Epidermal Growth Factor Receptor (EGFR)-targeting peptide and tested for their enhanced cellular uptake in human colon cancer cell lines as well tumor targeting ability *in vivo*. In another study, P(3HB-3HHx) was fabricated into nanoparticles for encapsulation of several hydrophobic kinase inhibitors (Lu et al., 2010). It was observed that the drug-loaded P(3HB-3HHx) NPs achieved higher drug loading efficiency as well as better sustained release than polylactic acid (PLA) NPs. PHA particles containing Rhodamine B Isothiocyanate along with a targeting moiety consisting of the PhaP protein fused with mannosylated human glycoprotein and human epidermal growth factor, specifically recognize and bind macrophages and hepatocellular carcinoma cells thus achieving targeted drug delivery (Yao et al., 2008). This illustrated how bacterial polymers could turn into an intelligent system for cell targeting purposes.

Inclusion bodies, that holds biomedically useful macromolecules such as PHA have long been considered undesired byproducts. Recombinant protein production in bacterial micro-factories do not always function in a physiologically perfect fashion and causes accumulation of insoluble protein aggregates inside the cell that never reach their native conformation (Gasser et al., 2008). These protein aggregates, also known as inclusion bodies, ranging in size from 200 to 500 nm, have been characterized as self-organized, stable bodies (Margreiter et al., 2008), that being fully biocompatible, can be used, for instance, in tissue engineering and regenerative medicine. In addition, they are now being recognized as biologically functional materials, and using genetic approaches like for example, inactivation of specific genes determining cell quality control, such as dnaK encoding a chaperone or clpP encoding a protease, inclusion bodies offer an interesting platform for tailored biofabrication of nanosystems for biomedical applications (García-Fruitós et al., 2010).

Microbial polysaccharides particularly, exopolysaccharides (EPSs) have drawn immense potential in biomaterial science as a source for fabrication of colloidal-based nanoparticles. Besides their functional role in biofilm formation and as defense tools against abiotic and biotic environmental stress (Poli et al., 2011), EPSs are good metal chelators and functionalized polymers, where various metal ions tend to get reduced and stabilized in colloidal state nanoparticles. Microorganisms produce these polysaccharides in enough quantities when the reactor culture is kept under limiting conditions of nitrogen or oxygen with excess of carbon source in the growth environment (Poli et al., 2011; Roca et al., 2015). A number of structurally known EPS like succinoglycan, dextran as nanoparticle capping or stabilizing agent, xanthan gum for the stabilization and synthesis of AuNPs, FeNPs, and PdNPs, Mauran from an extremophilic bacteria, their mechanism of NP synthesis has been well-reported (Sathiyanarayanan et al., 2017). AuNPs were prepared by EPS, extracted from *Lactobacillus plantarum* and functionalized with multiple antibiotics to treat multidrug resistant (MDR) bacterial infections (Pradeepa et al., 2016).

Dextrans and derivatives produced from *Leuconostoc sp*. growing on sucrose, *Streptococcus, Lactobacillus*, and *Gluconobacter sp* have been used in controlled release system in nasal and colon targeting (Mellors et al., 2010; Huang and Huang, 2019). Xanthan is another anionic and a naturally acetylated cellulose derivative from *Xanthomonas campestris*. It is an industrially popular biomolecule available since 1964; also, been used as an effective drug carrier and *in situ* bioadhesive nasal insert for extended drug delivery (Becker and Vorhoelter, 2009). Similarly, Gellan gum is an anionic but deacetylated exopolysaccharide obtained from *Sphingomonas paucimobilis*; has a characteristic temperature and ion dependent gelling property, is suitable for controlled release of antibiotics against stomach ulcer (Rajinikanth and Mishra, 2009). It showed long lasting stability and sustained release over an 8 H period. A multifunctional magnetic nanoparticle coated with two bacterial EPS-Mauran and Gellan Gum along with a targeting moiety, folate encapsulating a potent anticancer drug, 5-fluorouracil (5-FU) was developed (Sivakumar et al., 2014). More than 80% of cancer cells was killed within 60 min of magnetic hyperthermia application. A biopolymer (Chitosan) - bacterial polysaccharide complexed nanoparticulate formulation was synthesized using Mauran, the sulfated EPS extracted from a halophilic bacterium, *Halomonas maura* (Raveendran et al., 2013). The nanoparticles worked as a pH sensitive drug release system with a sustained and prolonged delivery of 5-FU for more than 10 days under three different pHs of 4.5, 6.9, and 7.4. Chitosan based nanostructures are popular drug delivery agents due to its biodegradability, biocompatibility, and its ability to form hydrogel. El Sherbiny et al. have demonstrated green synthesis of Silver (Ag) and Gold (Au) hybrid nanoparticles with Chitosan and a plant extract as reducing and stabilizing/capping agent (El-Sherbiny and Sedki, 2019). Gold nanoparticles (AuNPs) of fungal origin using microbial glycolipid mannosylerythritol lipid (MEL) produced from *Ustilago maydis* was biofabricated with potential biomedical activities against HepG2 cells and inhibited the growth of gram-positive and gram-negative pathogens (Bakur et al., 2019).

Although alginates are mostly extracted from brown seaweeds, some tailor-made acetylated alginates produced by *Azotobacter* and *Pseudomonas* genera have been applied as a dressing in surgery and wound management or matrix for the entrapment and delivery of proteins and cells (Remminghorst and Rehm, 2009). Hyaluronic acid (HA) is an extracellular capsular biopolymer synthesized by Lancefield group A and C *Streptococci* (Izawa et al., 2011). HA-based nanoformulations have been explored in formulating drug delivery systems and as hydrogel matrix in tissue engineering on account of its viscoelastic properties, its ability to inhibit platelet adhesion, and a complete lack of immunogenicity. The self-assembling nanoparticles served as a long-lasting ocular delivery product (Ibrahim et al., 2010) and also in the intravenous delivery of antitumor drugs, proteins, or nucleic acids (Ossipov, 2010).

Iron Oxide NPs, due to their high surface energy tend to aggregate rapidly in aqueous media. When combined with bacterial nanocellulose, such metal oxide NPs show improved dispersion as the polysaccharide provides an excellent biocompatible shell to tackle the issue of aggregation and delay the immune recognition too. Several bacteria and fungi such as *Komagataeibacter, Agrobacterium, Pseudomonas, Rhizobium*, or *Alcaligenes* secrete nanofibrillar cellulose as an exo-polysaccharide (Gorgieva and Trček, 2019). These biosynthesized cellulose bear higher crystallinity, purity, and water uptake capability besides biodegradability (Oprea and Panaitescu, 2020).

Several poly-(amino acids) like poly (γ-glutamic acid) (γ-PGA) are produced by bacteria, nanoparticles of which has been used as efficient drug carriers of erythromycin and α-chymotrypsin (Portilla-Arias et al., 2009). Poly (ε-lactides) (ε-PL) is a linear cationic polymer secreted by some of the *Streptomyces* strains (Saimura et al., 2008), have been applied as high-water retaining hydrogels in drug delivery and biosensing devices. In addition, poly ion complex nanoparticles with composition of biodegradable poly (gamma-glutamic acid) L-phenylalanine (γ-PGA-Phe) and poly(epsilon-lysine) (ε-PL) were tested under physiological conditions as multifunctional carriers for drug and vaccine delivery (Akagi et al., 2010).

Multi-L-arginyl-poly (L-aspartic acid) also called Cyanophycin (cyanobacterial origin), is a polymer of aspartic acid backbone, with arginine residues linked to the β-carboxyl group of each aspartic acid. It generally accumulates as cytoplasmic inclusions in most of the cyanobacterial cells and various chemical, enzymatic manipulations have resulted in derivatives with high biomedical potential (Lockau and Ziegler, 2006).

Thus, the basic advantage of bio-assisted fabrication of nanomaterials lie in their flexibility to modulate the quality of final product through organism-level biotechnological interventions or by simply changing the different conditions under which bacterial cells are cultivated. The wide diversity in microbial processing of metals, development of innovative nano-products, both inert and functionalized, not only provide solid proof of concept evidence of an emerging tool in basic nanomedicine, including tissue engineering, imaging and cancer therapy. The combination of some unique derivatization of microbial polymers along with properties attained at nanoscale have made such materials especially suitable to fill gaps in some of the most pressing needs of developing modern day nanomedicines.

## Conclusion Remarks and Future Outlook

Microbially derived metal nanoparticles are highly organized structures comprised of metal atoms or metal oxides with diverse applications in nanotechnology. The article presented a focused review on biosynthesis of metal nanoformulations by different microbial groups and their biomedical applications as a drug delivery and targeting agents. The potential of nano-organics derived from microbial cells have also been explored as a stabilizing and solubilizing agent besides their ability to allow functionalization on their surface. Bacterial magnetic nanoparticles are popular among biomedical researchers in their obvious utility as passive delivery agents of drug/gene fragments under an external magnetic field. Apart from several nanoformulations derived from bacteria, yeast, fungi, algae, the unique amorphous siliceous frustules of diatoms found important applications in drug delivery as well as in biotemplating.

Although naturally occurring microorganisms have been widely employed for the synthesis of several metal nanoparticles, their efficiency remains quite low; the size and composition of nanoparticles need to be better regulated. The limitation might also be attributed to implications of metal toxicity in living systems. Recently, scientists have called for act of balancing between positive therapeutic effects of metal oxide nanoparticles and their toxic side effects (Nikolova and Chavali, 2020). Long term toxicity may be induced due to delayed elimination, absence of dissolution/biodegradation followed by generation of intercellular reactive oxygen species (ROS), DNA damage, and triggered apoptotic cell death (Saptarshi et al., 2015; Shevtsov et al., 2018).

The global nanomaterials market is estimated to grow at a compounded annual growth rate (CAGR) of ~20% during the period 2016–22. With regard to clinical applicability and acceptability by the regulatory authorities, it should be noted that so far only three metal-based particles have been approved by the US FDA as per a report published in 2016 (Bobo et al., 2016). While majority of FDA-approved nanomedicines rely on passive targeting either *via* the EPR effect or under magnetic hyperthermia, there is a clear trend in emerging studies toward development of more complex functionalized systems for active targeting. This would further increase drug accumulation and ultimately bring about efficacy in the therapeutic potential at the disease site, while reducing reach in other organs.

The concept of “green chemistry” (design and synthesis of industrially viable products using environmentally sustainable precursors) synthesis and “white biotechnology” (harnessing the potential of living cells to synthesize products at industrial scale) when combined together, can significantly contribute to development of more sustainable industrial processes. Biotechnological approaches could allow screening of those gene sequences responsible for nanoparticle synthesis and a possible heterologous expression might enhance nanomaterial production efficiency. For instance, recombinant *E. coli* strain co-expressing enzymes metallothionein and phytochelatin synthase was constructed and employed as a highly efficient model host microorganism to biosynthesize as many as 60 different nanomaterials in both *in vitro* and *in vivo* conditions (Choi et al., 2018). The strategy allowed biogenic synthesis of nanomaterials with various properties, providing a platform for manufacturing different nanomaterials in an ecologically friendly manner. However, microbial synthesis still cannot compete with chemical synthesis as the yield might not exceed by 1/3rd (Ovais et al., 2018). Despite the limitations, harnessing microorganisms for production of metal nanoparticles is very much favored considering the elimination of organic solvents, chemical stabilizing agents, and other environmental concerns during their synthesis.

## Author Contributions

All authors listed have made a substantial, direct and intellectual contribution to the work, and approved it for publication.

## Conflict of Interest

The authors declare that the research was conducted in the absence of any commercial or financial relationships that could be construed as a potential conflict of interest.

## References

[B1] Aggarwal V. Malik J. Prashant A. Jaiwal P. K. Pundir C. S. (2016). Amperometric determination of serum total cholesterol with nanoparticles of cholesterol esterase and cholesterol oxidase. Anal. Biochem. 500, 6–11. 10.1016/j.ab.2016.01.019 26853742

[B2] Akagi T. Watanabe K. Kim H. Akashi M. (2010). Stabilization of polyion complex nanoparticles composed of poly(amino acid) using hydrophobic interactions. Langmuir ACS J. Surf. Colloid. 26, 2406–2413. 10.1021/la902868g 20017513

[B3] AlNadhari S. Al-Enazi N. M. Alshehrei F. Ameen F. (2020). A review on biogenic synthesis of metal nanoparticles using marine algae and its applications. Environ. Res. 194:110672. 10.1016/j.envres.2020.110672 33373611

[B4] Alsaiari S. K. Ezzedine A. H. Abdallah A. M. Sougrat R. Khashab N. M. (2016). Magnetotactic bacterial cages as safe and smart gene delivery vehicles. OpenNano 1, 36–45. 10.1016/j.onano.2016.07.001

[B5] Ashraf N. Ahmad F. Da-Wei L. Zhou R. B. Feng-Li H. Yin D. C. (2019). Iron/iron oxide nanoparticles: advances in microbial fabrication, mechanism study, biomedical, and environmental applications. Crit. Rev. Microbiol. 45, 278–300. 10.1080/1040841X.2019.1593101 30985230

[B6] Bai H. J. Zhang Z. M. Guo Y. Yang G. E. (2009). Biosynthesis of cadmium sulfide nanoparticles by photosynthetic bacteria *Rhodopseudomonas palustris*. Colloid. Surf. B Biointerfaces 70, 142–146. 10.1016/j.colsurfb.2008.12.025 19167198

[B7] Bakur A. Niu Y. Kuang H. Chen Q. (2019). Synthesis of gold nanoparticles derived from mannosylerythritol lipid and evaluation of their bioactivities. AMB Express 9:62. 10.1186/s13568-019-0785-6 31065818 PMC6505018

[B8] Becker A. Vorhoelter F. J. (2009). “Xanthan biosynthesis by Xanthomonas bacteria: an overview of the current biochemical and genomic data,” in Microbial Production of Biopolymers and Polymer Precursors: Applications and Perspectives, ed B. Rehm (Poole, UK: Caister Academic Press), 1–11.

[B9] Bobo D. Robinson K. J. Islam J. Thurecht K. J. Corrie S. R. (2016). Nanoparticle-based medicines: a review of FDA-approved materials and clinical trials to date. Pharm. Res. 33, 2373–2387. 10.1007/s11095-016-1958-5 27299311

[B10] Bulgarini A. Lampis S. Turner R. J. Vallini G. (2020). Biomolecular composition of capping layer and stability of biogenic selenium nanoparticles synthesized by five bacterial species. Microb. Biotechnol. 14, 198–212. 10.1111/1751-7915.13666 33068075 PMC7888468

[B11] Chao J. T. Biggs M. J. Pandit A. S. (2014). Diatoms: a biotemplating approach to fabricating drug delivery reservoirs. Expert Opin. Drug Deliv. 11, 1687–1695. 10.1517/17425247.2014.935336 25146231

[B12] Chauhan N. Kumar A. Pundir C. S. (2014). Construction of an uricase nanoparticles modified au electrode for amperometric determination of uric acid. Appl. Biochem. Biotechnol. 174, 1683–1694. 10.1007/s12010-014-1097-6 25141984

[B13] Cheng L. Ke Y. Yu S. Jing J. (2016). Co-delivery of doxorubicin and recombinant plasmid pHSP70-Plk1-shRNA by bacterial magnetosomes for osteosarcoma therapy. Int. J. Nanomed. 11, 5277–5286. 10.2147/IJN.S115364 27822032 PMC5087786

[B14] Choi Y. Park T. J. Lee D. C. Lee S. Y. (2018). Recombinant *Escherichia coli* as a biofactory for various single- and multi-element nanomaterials. Proc. Natl. Acad. Sci. U. S. A. 115, 5944–5949. 10.1073/pnas.1804543115 29784775 PMC6003371

[B15] Cobbett C. Goldsbrough P. (2002). Phytochelatins and metallothioneins: roles in heavy metal detoxification and homeostasis. Annu. Rev. Plant Biol. 53, 159–182. 10.1146/annurev.arplant.53.100301.135154 12221971

[B16] Crichton R. (ed.). (2019). “Biomineralization” in Biological Inorganic Chemistry: A New Introduction to Molecular Structure and Function, 3rd Edn., (Amsterdam: Elsevier), 517–544.

[B17] Dai Q. Long R. Wang S. Kankala R. K. Wang J. Jiang W.. (2017). Bacterial magnetosomes as an efficient gene delivery platform for cancer theranostics. Microb. Cell Fact 16:216. 10.1186/s12934-017-0830-6 29183380 PMC5704436

[B18] Daneshpour M. Karimi B. Omidfar K. (2018). Simultaneous detection of gastric cancer-involved miR-106a and let-7a through a dual-signal-marked electrochemical nanobiosensor. Biosens. Bioelectron. 109, 197–205. 10.1016/j.bios.2018.03.022 29567564

[B19] Delasoie J. Zobi F. (2019). Natural diatom biosilica as microshuttles in drug delivery systems. Pharmaceutics 11:537. 10.3390/pharmaceutics11100537 31618958 PMC6835591

[B20] Dhanalekshmi K. I. Sangeetha K. Magesan P. Johnson J. Zhang X. Jayamoorthy K. (2020). Photodynamic cancer therapy: role of Ag- and Au-based hybrid nano-photosensitizers. J. Biomol. Struct. Dyn. 1–8. 10.1080/07391102.2020.1858965. [Epub ahead of print]. 33300461

[B21] El-Sherbiny I. M. Sedki M. (2019). Green synthesis of chitosan-silver/gold hybrid nanoparticles for biomedical applications. Methods Mol. Biol. 2000, 79–84. 10.1007/978-1-4939-9516-5_7 31148010

[B22] Erdal E. Demirbilek M. Yeh Y. Akbal Ö. Ruff L. Bozkurt D.. (2018). A comparative study of receptor-targeted magnetosome and HSA-coated iron oxide nanoparticles as MRI contrast-enhancing agent in animal cancer model. Appl. Biochem. Biotechnol. 185, 91–113. 10.1007/s12010-017-2642-x 29082480

[B23] Fahmy S. A. Preis E. Bakowsky U. Azzazy H. (2020). Platinum nanoparticles: green synthesis and biomedical applications. Molecules 25:4981. 10.3390/molecules25214981 33126464 PMC7662215

[B24] Fan F. Wang L. Ouyang Z. Wen Y. Lu X. (2018). Development and optimization of a tumor targeting system based on microbial synthesized PHA biopolymers and PhaP mediated functional modification. Appl. Microbiol. Biotechnol. 102, 3229–3241. 10.1007/s00253-018-8790-2 29497797

[B25] Felfoul O. Mohammadi M. Taherkhani S. de Lanauze D. Zhong Xu Y. Loghin D.. (2016). Magneto-aerotactic bacteria deliver drug-containing nanoliposomes to tumour hypoxic regions. Nat. Nanotechnol. 11, 941–947. 10.1038/nnano.2016.137 27525475 PMC6094936

[B26] Firlar E. Ouy M. Bogdanowicz A. Covnot L. Song B. Nadkarni Y.. (2019). Investigation of the magnetosome biomineralization in magnetotactic bacteria using graphene liquid cell - transmission electron microscopy. Nanoscale 11, 698–705. 10.1039/C8NR08647H 30565643

[B27] Gadd G. M. Pan X. (2016). Biomineralization, bioremediation and biorecovery of toxic metals and radionuclides. Geomicrobiol. J. 33, 175–178. 10.1080/01490451.2015.1087603

[B28] Gandia D. Gandarias L. Rodrigo I. Robles-García J. Das R. Garaio E.. (2019). Unlocking the potential of magnetotactic bacteria as magnetic hyperthermia agents. Small 15:e1902626. 10.1002/smll.201902626 31454160

[B29] García-Fruitós E. Seras-Franzoso J. Vazquez E. Villaverde A. (2010). Tunable geometry of bacterial inclusion bodies as substrate materials for tissue engineering. Nanotechnology 21:205101. 10.1088/0957-4484/21/20/205101 20413834

[B30] Gasser B. Saloheimo M. Rinas U. Dragosits M. Rodríguez-Carmona E. Baumann K.. (2008). Protein folding and conformational stress in microbial cells producing recombinant proteins: a host comparative overview. Microb. Cell Fact 7:11. 10.1186/1475-2859-7-11 18394160 PMC2322954

[B31] Geng Y. Wang J. Wang X. Liu J. Zhang Y. Niu W.. (2019). Growth-inhibitory effects of anthracycline-loaded bacterial magnetosomes against hepatic cancer *in vitro* and *in vivo*. Nanomedicine 14, 1663–1680. 10.2217/nnm-2018-0296 31167626

[B32] Gorgieva S. Trček J. (2019). Bacterial cellulose: production, modification and perspectives in biomedical applications. Nanomaterials 9:1352. 10.3390/nano9101352 31547134 PMC6835293

[B33] Grachev M. A. Annenkov V. V. Likhoshway Y. V. (2008). Silicon nanotechnologies of pigmented heterokonts. BioEssays News Rev. Mol. Cell. Dev. Biol. 30, 328–337. 10.1002/bies.20731 18348175

[B34] Grasso G. Zane D. Dragone R. (2019). Microbial nanotechnology: challenges and prospects for green biocatalytic synthesis of nanoscale materials for sensoristic and biomedical applications. Nanomaterials 10:11. 10.3390/nano10010011 31861471 PMC7023511

[B35] Hamouda R. A. Hussein M. H. Abo-Elmagd R. A. Bawazir S. S. (2019). Synthesis and biological characterization of silver nanoparticles derived from the cyanobacterium *Oscillatoria limnetica*. Sci. Rep. 9:13071. 10.1038/s41598-019-49444-y 31506473 PMC6736842

[B36] Harada L. K. Silva E. C. Campos W. F. Del Fiol F. S. Vila M. Dabrowska K.. (2018). Biotechnological applications of bacteriophages: state of the art. Microbiol. Res. 212–213, 38–58. 10.1016/j.micres.2018.04.007 29853167

[B37] Hathout R. M. Metwally A. A. (2019). Gelatin nanoparticles. Methods Mol. Biol. 2000, 71–78. 10.1007/978-1-4939-9516-5_6 31148009

[B38] Hu X. Saravanakumar K. Jin T. Wang M. H. (2019). Mycosynthesis, characterization, anticancer and antibacterial activity of silver nanoparticles from endophytic fungus *Talaromyces purpureogenus*. Int. J. Nanomed. 14, 3427–3438. 10.2147/IJN.S200817 31190801 PMC6515543

[B39] Huang J. Y. Chen M. H. Lin F. H. (2019). The synthesis and characterization of PEG-SH-modified gold nanoparticle in one-pot synthesis by *Stenotrophomonas maltophilia*. J. Nanosci. Nanotechnol. 19, 7278–7284. 10.1166/jnn.2019.16625 31039886

[B40] Huang M. Li T. Pan T. Zhao N. Yao Y. Zhai Z.. (2016). Controlling the strontium-doping in calcium phosphate microcapsules through yeast-regulated biomimetic mineralization. Regen. Biomater. 3, 269–276. 10.1093/rb/rbw025 27699057 PMC5043151

[B41] Huang S. Huang G. (2019). The dextrans as vehicles for gene and drug delivery. Future Med. Chem. 11, 1659–1667. 10.4155/fmc-2018-0586 31469330

[B42] Ibrahim H. K. El-Leithy I. S. Makky A. A. (2010). Mucoadhesive nanoparticles as carrier systems for prolonged ocular delivery of gatifloxacin/prednisolone bitherapy. Mol. Pharm. 7, 576–585. 10.1021/mp900279c 20163167

[B43] Izawa N. Serata M. Sone T. Omasa T. Ohtake H. (2011). Hyaluronic acid production by recombinant Streptococcus thermophilus. J. Biosci. Bioeng. 111, 665–670. 10.1016/j.jbiosc.2011.02.005 21371932

[B44] Jin W. Yu Y. Hou W. Wang G. Zhu Z. He J.. (2019). Molecular characteristics of kappa-selenocarrageenan and application in green synthesis of silver nanoparticles. Int. J. Biol. Macromol. 141, 529–537. 10.1016/j.ijbiomac.2019.09.016 31493457

[B45] Kabir A. Nazeer N. Bissessur R. Ahmed M. (2020). Diatoms embedded, self-assembled carriers for dual delivery of chemotherapeutics in cancer cell lines. Int. J. Pharm. 573:118887. 10.1016/j.ijpharm.2019.118887 31765771

[B46] Kalishwaralal K. Banumathi E. Ram Kumar Pandian S. Deepak V. Muniyandi J. Eom S. H.. (2009). Silver nanoparticles inhibit VEGF induced cell proliferation and migration in bovine retinal endothelial cells. Colloid. Surf. B Biointerfaces 73, 51–57. 10.1016/j.colsurfb.2009.04.025 19481908

[B47] Kaspar A. A. Reichert J. M. (2013). Future directions for peptide therapeutics development. Drug Discov. Today 18, 807–817. 10.1016/j.drudis.2013.05.011 23726889

[B48] Kikuchi F. Kato Y. Furihata K. Kogure T. Imura Y. Yoshimura E.. (2016). Formation of gold nanoparticles by glycolipids of Lactobacillus casei. Sci. Rep. 6:34626. 10.1038/srep34626 27725710 PMC5057074

[B49] Kotelnikova P. A. Shipunova V. O. Aghayeva U. F. Stremovskiy O. A. Nikitin M. P. Novikov I. A.. (2018). Synthesis of magnetic nanoparticles stabilized by magnetite-binding protein for targeted delivery to cancer cells. Doklady Biochem. Biophys. 481, 198–200. 10.1134/S1607672918040051 30168058

[B50] Kratošová G. Holišová V. Konvičková Z. Ingle A. P. Gaikwad S. Škrlová K.. (2019). From biotechnology principles to functional and low-cost metallic bionanocatalysts. Biotechnol. Adv. 37, 154–176. 10.1016/j.biotechadv.2018.11.012 30481544

[B51] Kumar K. Kumar Doddi S. Arunasree M. K. Paik P. (2015). CPMV-induced synthesis of hollow mesoporous SiO_2_ nanocapsules with excellent performance in drug delivery. Dalton Trans. 44, 4308–4317. 10.1039/C4DT02549K 25640798

[B52] Kumeria T. Bariana M. Altalhi T. Kurkuri M. Gibson C. T. Yang W.. (2013). Graphene oxide decorated diatom silica articles as new nano-hybrids: towards smart natural drug microcarriers. J. Mater. Chem. B, 1, 6302–6311. 10.1039/c3tb21051k 32261703

[B53] Kundu N. Yadav S. Pundir C. S. (2013). Preparation and characterization of glucose oxidase nanoparticles and their application in dissolved oxygen metric determination of serum glucose. J. Nanosci. Nanotechnol. 13, 1710–1716. 10.1166/jnn.2013.7102 23755578

[B54] Kuzajewska D. Wszołek A. Zwierełło W. Kirczuk L. Maruszewska A. (2020). Magnetotactic bacteria and magnetosomes as smart drug delivery systems: a new weapon on the battlefield with cancer? Biology 9:102. 10.3390/biology9050102 32438567 PMC7284773

[B55] Le T. C. Yin H. Chen R. Chen Y. Zhao L. Casey P. S.. (2016). An experimental and computational approach to the development of ZnO nanoparticles that are safe by design. Small 12, 3568–3577. 10.1002/smll.201600597 27167706

[B56] Lechner C. C. Becker C. F. (2015). Silaffins *in silica* biomineralization and biomimetic silica precipitation. Mar. Drugs 13, 5297–5333. 10.3390/md13085297 26295401 PMC4557024

[B57] Li J. Webster T. J. Tian B. (2019). Functionalized nanomaterial assembling and biosynthesis using the extremophile *Deinococcus radiodurans* for multifunctional applications. Small 15:e1900600. 10.1002/smll.201900600 30925017

[B58] Li N. Shang Y. Han Z. Wang T. Wang Z. G. Ding B. (2019). Fabrication of metal nanostructures on DNA templates. ACS Appl. Mater. Interfaces 11, 13835–13852. 10.1021/acsami.8b16194 30480424

[B59] Li T. Hao L. Du C. Wang Y. (2017). Synthesis of magnesium-doped calcium carbonate microcapsules through yeast-regulated mineralization. Mater. Lett. 193, 38–41. 10.1016/j.matlet.2017.01.093

[B60] Li Z. Loh X. J. (2015). Water soluble polyhydroxyalkanoates: future materials for therapeutic applications. Chem. Soc. Rev. 44, 2865–2879. 10.1039/C5CS00089K 25788317

[B61] Liu G. Lin Y. Ostatn,á V. Wang J. (2005). Enzyme nanoparticles-based electronic biosensor. Chem. Commun. 27, 3481–3483. 10.1039/b504943a 15997304

[B62] Liu Y. Perumalsamy H. Kang C. H. Kim S. H. Hwang J. S. Koh S. C.. (2020). Intracellular synthesis of gold nanoparticles by *Gluconacetobacter liquefaciens* for delivery of peptide CopA3 and ginsenoside and anti-inflammatory effect on lipopolysaccharide-activated macrophages. Artificial Cell. Nanomed. Biotechnol. 48, 777–788. 10.1080/21691401.2020.1748639 32308043

[B63] Lockau W. Ziegler K. (2006). “Cyanophycin inclusions: biosynthesis and applications,” in Microbial Bionanotechnology - Biological Self-assembly Systems and Biopolymer-based Nanostructures, ed B. Rehm (Wymondham, UK: Horizon Bioscience) 78–106.

[B64] Long R. Liu Y. Dai Q. Wang S. Deng Q. Zhou X. (2016). A natural bacterium-produced membrane-bound nanocarrier for drug combination therapy. Materials 9:889. 10.3390/ma9110889 28774010 PMC5457273

[B65] Losic D. Yu Y. Aw M. S. Simovic S. Thierry B. Addai-Mensah J. (2010). Surface functionalisation of diatoms with dopamine modified iron-oxide nanoparticles: toward magnetically guided drug microcarriers with biologically derived morphologies. Chem. Commun. 46, 6323–6325. 10.1039/c0cc01305f 20676447

[B66] Lu X.-Y. Zhang Y. Wang L. (2010). Preparation and *in vitro* drug-release behavior of 5-fluorouracil-loaded poly(hydroxybutyrate-*co*-hydroxyhexanoate) nanoparticles and microparticles. J. Appl. Polym. Sci. 116, 2944–2950. 10.1002/app.31806

[B67] Ma X. Liu P. Tian Y. Zhu G. Yang P. Wang G.. (2018). A mineralized cell-based functional platform: construction of yeast cells with biogenetic intracellular hydroxyapatite nanoscaffolds. Nanoscale 10, 3489–3496. 10.1039/C7NR07714A 29404549

[B68] Maher S. Kumeria T. Aw M. S. Losic D. (2018). Diatom silica for biomedical applications: recent progress and advances. Adv. Healthc. Mater. 7:e1800552. 10.1002/adhm.201800552 30118185

[B69] Maher S. Santos A. Kumeria T. Kaur G. Lambert M. Forward P.. (2017). Multifunctional microspherical magnetic and pH responsive carriers for combination anticancer therapy engineered by droplet-based microfluidics. J. Mater. Chem. B 5, 4097–4109. 10.1039/C7TB00588A 32264142

[B70] Margreiter G. Messner P. Caldwell K. D. Bayer K. (2008). Size characterization of inclusion bodies by sedimentation field-flow fractionation. J. Biotechnol. 138, 67–73. 10.1016/j.jbiotec.2008.07.1995 18760314 PMC4388406

[B71] Martucci N. M. Migliaccio N. Ruggiero I. Albano F. Calì G. Romano S.. (2016). Nanoparticle-based strategy for personalized B-cell lymphoma therapy. Int. J. Nanomed. 11, 6089–6101. 10.2147/IJN.S118661 27895482 PMC5117954

[B72] Mellors R. Benzeval I. Eisenthal R. Hubble J. (2010). Preparation of self-assembled microspheres and their potential for drug delivery. Pharm. Dev. Technol. 15, 105–111. 10.3109/10837450903036163 19545194

[B73] Narayanan K. B. Sakthivel N. (2010). Biological synthesis of metal nanoparticles by microbes. Adv. Colloid Interface Sci. 156, 1–13. 10.1016/j.cis.2010.02.001 20181326

[B74] Nikolova M. P. Chavali M. S. (2020). Metal oxide nanoparticles as biomedical materials. Biomimetics 5:27. 10.3390/biomimetics5020027 32521669 PMC7345077

[B75] Nima Z. A. Watanabe F. Jamshidi-Parsian A. Sarimollaoglu M. Nedosekin D. A. Han M.. (2019). Bioinspired magnetic nanoparticles as multimodal photoacoustic, photothermal and photomechanical contrast agents. Sci. Rep. 9:887. 10.1038/s41598-018-37353-5 30696936 PMC6351522

[B76] Oprea M. Panaitescu D. M. (2020). Nanocellulose hybrids with metal oxides nanoparticles for biomedical applications. Molecules 25:4045. 10.3390/molecules25184045 32899710 PMC7570792

[B77] Ossipov D. A. (2010). Nanostructured hyaluronic acid-based materials for active delivery to cancer. Expert Opin. Drug Deliv. 7, 681–703. 10.1517/17425241003730399 20367530

[B78] Ovais M. Khalil A. T. Ayaz M. Ahmad I. Nethi S. K. Mukherjee S. (2018). Biosynthesis of metal nanoparticles *via* microbial enzymes: a mechanistic approach. Int. J. Mol. Sci. 19:4100. 10.3390/ijms19124100 30567324 PMC6321641

[B79] Park T. J. Lee K. G. Lee S. Y. (2016). Advances in microbial biosynthesis of metal nanoparticles. Appl. Microbiol. Biotechnol. 100, 521–534. 10.1007/s00253-015-6904-7 26300292

[B80] Patil M. P. Kim G. D. (2018). Marine microorganisms for synthesis of metallic nanoparticles and their biomedical applications. Colloid. Surf. B Biointerfaces 172, 487–495. 10.1016/j.colsurfb.2018.09.007 30205339

[B81] Petrenko V. A. Jayanna P. K. (2014). Phage protein-targeted cancer nanomedicines. FEBS Lett. 588, 341–349. 10.1016/j.febslet.2013.11.011 24269681 PMC4557960

[B82] Poli A. Di Donato P. Abbamondi G. R. Nicolaus B. (2011). Synthesis, production, and biotechnological applications of exopolysaccharides and polyhydroxyalkanoates by archaea. Archaea 2011:693253. 10.1155/2011/693253 22007151 PMC3191746

[B83] Portilla-Arias J. A. Camargo B. García-Alvarez M. de Ilarduya A. M. Muñoz-Guerra S. (2009). Nanoparticles made of microbial poly(gamma-glutamate)s for encapsulation and delivery of drugs and proteins. J. Biomater. Sci. Polym. 20, 1065–1079. 10.1163/156856209X444420 19454169

[B84] Poulose S. Panda T. Nair P. P. Théodore T. (2014). Biosynthesis of silver nanoparticles. J. Nanosci. Nanotechnol. 14, 2038–2049. 10.1166/jnn.2014.9019 24749472

[B85] Pradeepa Vidya S. M. Mutalik S. Udaya Bhat K. Huilgol P. Avadhani K. (2016). Preparation of gold nanoparticles by novel bacterial exopolysaccharide for antibiotic delivery. Life Sci. 153, 171–179. 10.1016/j.lfs.2016.04.022 27101926

[B86] Pumpens P. Renhofa R. Dishlers A. Kozlovska T. Ose V. Pushko P.. (2016). The true story and advantages of RNA phage capsids as nanotools. Intervirology 59, 74–110. 10.1159/000449503 27829245

[B87] Qin W. Wang C. Y. Ma Y. X. Shen M. J. Li J. Jiao K.. (2020). Microbe-mediated extracellular and intracellular mineralization: environmental, industrial, and biotechnological applications. Adv. Mater. 32:e1907833. 10.1002/adma.201907833 32270552

[B88] Rajeswaran S. Somasundaram Thirugnanasambandan S. Dewangan N. K. Moorthy R. K. Kandasamy S. Vilwanathan R. (2020). Multifarious pharmacological applications of green routed eco-friendly iron nanoparticles synthesized by *Streptomyces sp*. (SRT12). Biol. Trace Element Res. 194, 273–283. 10.1007/s12011-019-01777-5 31256390

[B89] Rajinikanth P. S. Mishra B. (2009). Stomach-site specific drug delivery system of clarithromycin for eradication of *Helicobacter pylori*. Chem. Pharm. Bull. 57, 1068–1075. 10.1248/cpb.57.1068 19801860

[B90] Ramesh S. Grijalva M. Debut A. de la Torre B. G. Albericio F. Cumbal L. H. (2016). Peptides conjugated to silver nanoparticles in biomedicine - a “value-added” phenomenon. Biomater. Sci. 4, 1713–1725. 10.1039/C6BM00688D 27748772

[B91] Raveendran S. Poulose A. C. Yoshida Y. Maekawa T. Kumar D. S. (2013). Bacterial exopolysaccharide based nanoparticles for sustained drug delivery, cancer chemotherapy and bioimaging. Carbohydr. Polym. 91, 22–32. 10.1016/j.carbpol.2012.07.079 23044101

[B92] Rehm B. H. Steinbüchel A. (1999). Biochemical and genetic analysis of PHA synthases and other proteins required for PHA synthesis. Int. J. Biol. Macromol. 25, 3–19. 10.1016/S0141-8130(99)00010-0 10416645

[B93] Remminghorst U. Rehm B. H. (2009). “Microbial production of alginate: biosynthesis and applications,” in Microbial Production of Biopolymers and Polymer Precursors: Applications and Perspectives, ed B. Rehm (Poole, UK: Caister Academic Press), 13–42.

[B94] Rhea I. Martucci N. M. De Stefano L. Ruggiero I. Terracciano M. Dardano P.. (2014). Diatomite biosilica nanocarriers for siRNA transport inside cancer cells. Biochim. Biophys. Acta 1840, 3393–3403. 10.1016/j.bbagen.2014.09.009 25224732

[B95] Rhea I. Terracciano M. De Stefano L. (2017). Synthetic vs. natural: diatoms bioderived porous materials for the next generation of healthcare nanodevices. Adv. Healthcare Mater. 6:1125. 10.1002/adhm.201601125 28026914

[B96] Rizzo L. Y. Theek B. Storm G. Kiessling F. Lammers T. (2013). Recent progress in nanomedicine: therapeutic, diagnostic and theranostic applications. Curr. Opin. Biotechnol. 24, 1159–1166. 10.1016/j.copbio.2013.02.020 23578464 PMC3833836

[B97] Roca C. Alves V. D. Freitas F. Reis M. A. (2015). Exopolysaccharides enriched in rare sugars: bacterial sources, production, and applications. Front. Microbiol. 6:288. 10.3389/fmicb.2015.00288 25914689 PMC4392319

[B98] Sable S. V. Kawade S. Ranade S. Joshi S. (2020). Bioreduction mechanism of silver nanoparticles. Mater. Sci. Eng. C Mater. Biol. Application. 107:110299. 10.1016/j.msec.2019.110299 31761186

[B99] Saeed B. A. Lim V. Yusof N. A. Khor K. Z. Rahman H. S. Abdul Samad N. (2019). Antiangiogenic properties of nanoparticles: a systematic review. Int. J. Nanomed. 14, 5135–5146. 10.2147/IJN.S199974 31371952 PMC6630093

[B100] Saimura M. Takehara M. Mizukami S. Kataoka K. Hirohara H. (2008). Biosynthesis of nearly monodispersed poly(epsilon-L-lysine) in *Streptomyces* species. Biotechnol. Lett. 30, 377–385. 10.1007/s10529-007-9563-7 17985083

[B101] Salem N. Abouelkheir S. S. Yousif A. M. Meneses-Brassea B. P. Sabry S. A. Ghozlan H. A.. (2021). Large scale production of superparamagnetic iron oxide nanoparticles by the haloarchaeon *Halobiforma* sp. N1 and their potential in localized hyperthermia cancer therapy. Nanotechnology 32:09LT01. 10.1088/1361-6528/abc851 33157540

[B102] Salunke B. K. Sawant S. S. Lee S. I. Kim B. S. (2016). Microorganisms as efficient biosystem for the synthesis of metal nanoparticles: current scenario and future possibilities. World J. Microbiol. Biotechnol. 32:88. 10.1007/s11274-016-2044-1 27038958

[B103] Saptarshi S. R. Duschl A. Lopata A. L. (2015). Biological reactivity of zinc oxide nanoparticles with mammalian test systems: an overview. Nanomedicine 10, 2075–2092. 10.2217/nnm.15.44 26135328

[B104] Saratale R. G. Karuppusamy I. Saratale G. D. Pugazhendhi A. Kumar G. Park Y.. (2018). A comprehensive review on green nanomaterials using biological systems: recent perception and their future applications. Colloid. Surf. B Biointerfaces 170, 20–35. 10.1016/j.colsurfb.2018.05.045 29860217

[B105] Sathiyanarayanan G. Dineshkumar K. Yang Y. H. (2017). Microbial exopolysaccharide-mediated synthesis and stabilization of metal nanoparticles. Crit. Rev. Microbiol. 43, 731–752. 10.1080/1040841X.2017.1306689 28440091

[B106] Schuerle S. Soleimany A. P. Yeh T. Anand G. M. Häberli M. Fleming H. E.. (2019). Synthetic and living micropropellers for convection-enhanced nanoparticle transport. Sci. Adv. 5:eaav4803. 10.1126/sciadv.aav4803 31032412 PMC6486269

[B107] Shakesheff K. M. Evora C. Soriano I. I. Langer R. (1997). The adsorption of poly(vinyl alcohol) to biodegradable microparticles studied by x-ray photoelectron spectroscopy (XPS). J. Colloid Interface Sci. 185, 538–547. 10.1006/jcis.1996.4637 9028908

[B108] Shevtsov M. Nikolaev B. Marchenko Y. Yakovleva L. Skvortsov N. Mazur A.. (2018). Targeting experimental orthotopic glioblastoma with chitosan-based superparamagnetic iron oxide nanoparticles (CS-DX-SPIONs). Int. J. Nanomed. 13, 1471–1482. 10.2147/IJN.S152461 29559776 PMC5856030

[B109] Singh A. K. Srivastava J. K. Chandel A. K. Sharma L. Mallick N. Singh S. P. (2019). Biomedical applications of microbially engineered polyhydroxyalkanoates: an insight into recent advances, bottlenecks, and solutions. Appl. Microbiol. Biotechnol. 103, 2007–2032. 10.1007/s00253-018-09604-y 30645689

[B110] Sivakumar B. Aswathy R. G. Sreejith R. Nagaoka Y. Iwai S. Suzuki M.. (2014). Bacterial exopolysaccharide based magnetic nanoparticles: a versatile nanotool for cancer cell imaging, targeted drug delivery and synergistic effect of drug and hyperthermia mediated cancer therapy. J. Biomed. Nanotechnol. 10, 885–899. 10.1166/jbn.2014.1820 24749386

[B111] Srivastava P. Bragança J. Ramanan S. R. Kowshik M. (2013). Synthesis of silver nanoparticles using haloarchaeal isolate *Halococcus salifodinae* BK3. Extremophiles Life Under Extreme Conditions 17, 821–831. 10.1007/s00792-013-0563-3 23884709

[B112] Tanaka T. Kokuryu Y. Matsunaga T. (2008). Novel method for selection of antimicrobial peptides from a phage display library by use of bacterial magnetic particles. Appl. Environ. Microbiol. 74, 7600–7606. 10.1128/AEM.00162-08 18952877 PMC2607169

[B113] Tang Y. S. Wang D. Zhou C. Zhang S. (2019). Preparation and anti-tumor efficiency evaluation of bacterial magnetosome-anti-4–1BB antibody complex: bacterial magnetosome as antibody carriers isolated from *Magnetospirillum gryphiswaldense*. Biotechnol. Appl. Biochem. 66, 290–297. 10.1002/bab.1724 30600567

[B114] Temple K. L. Le Roux N. W. (1964). Syngenesis of sulfide ores; desorption of adsorbed metal ions and their precipitation as sulfides. Econ. Geol. 59:647–665 10.2113/gsecongeo.59.4.647

[B115] Terracciano M. Napolitano M. De Stefano L. De Luca A. C. Rea I. (2018). Gold decorated porous biosilica nanodevices for advanced medicine. Nanotechnology 29:235601. 10.1088/1361-6528/aab7c4 29553482

[B116] Terracciano M. Shahbazi M. A. Correia A. Rea I. Lamberti A. De Stefano L.. (2015). Surface bioengineering of diatomite based nanovectors for efficient intracellular uptake and drug delivery. Nanoscale 7, 20063–20074. 10.1039/C5NR05173H 26568517

[B117] Todd T. Zhen Z. Tang W. Chen H. Wang G. Chuang Y. J.. (2014). Iron oxide nanoparticle encapsulated diatoms for magnetic delivery of small molecules to tumors. Nanoscale 6, 2073–2076. 10.1039/c3nr05623f 24424277 PMC3974590

[B118] Uthappa U. T. Brahmkhatri V. Sriram G. Jung H. Y. Yu J. Kurkuri N.. (2018). Nature engineered diatom biosilica as drug delivery systems. J. Control. Release 281, 70–83. 10.1016/j.jconrel.2018.05.013 29772290

[B119] Vaidyanathan R. Gopalram S. Kalishwaralal K. Deepak V. Pandian S. R. Gurunathan S. (2010). Enhanced silver nanoparticle synthesis by optimization of nitrate reductase activity. Colloids Surf. B Biointerf. 75, 335–341. 10.1016/j.colsurfb.2009.09.006 19796922

[B120] Vargas G. Cypriano J. Correa T. Leão P. Bazylinski D. A. Abreu F. (2018). Applications of magnetotactic bacteria, magnetosomes and magnetosome crystals in biotechnology and nanotechnology: mini-review. Molecules 23:2438. 10.3390/molecules23102438 30249983 PMC6222368

[B121] Wadhwani S. A. Shedbalkar U. U. Singh R. Chopade B. A. (2016). Biogenic selenium nanoparticles: current status and future prospects. Appl. Microbiol. Biotechnol. 100, 2555–2566. 10.1007/s00253-016-7300-7 26801915

[B122] Wang X. Liu X. Xiao Y. Hao H. Zhang Y. Tang R. (2018). Biomineralization state of viruses and their biological potential. Chemistry 24, 11518–11529. 10.1002/chem.201705936 29377301

[B123] Wnek M. Górzny M. L. Ward M. B. Wälti C. Davies A. G. Brydson R.. (2013). Fabrication and characterization of gold nano-wires templated on virus-like arrays of tobacco mosaic virus coat proteins. Nanotechnology 24:025605. 10.1088/0957-4484/24/2/025605 23220929 PMC4787025

[B124] Wuttke S. Lismont M. Escudero A. Rungtaweevoranit B. Parak W. J. (2017). Positioning metal-organic framework nanoparticles within the context of drug delivery—a comparison with mesoporous silica nanoparticles and dendrimers. Biomaterials 123, 172–183. 10.1016/j.biomaterials.2017.01.025 28182958

[B125] Xiang L. Bin W. Huali J. Wei J. Jiesheng T. Feng G.. (2007). Bacterial magnetic particles (BMPs)-PEI as a novel and efficient non-viral gene delivery system. J. Gene Med. 9, 679–690. 10.1002/jgm.1068 17605136

[B126] Yadav N. Narang J. Chhillar A. K. Pundir C. S. (2018). Preparation, characterization, and application of enzyme nanoparticles. Meth. Enzymol. 609, 171–196. 10.1016/bs.mie.2018.07.001 30244789

[B127] Yang F. Tang Q. Zhong X. Bai Y. Chen T. Zhang Y.. (2012). Surface decoration by *Spirulina polysaccharide* enhances the cellular uptake and anticancer efficacy of selenium nanoparticles. Int. J. Nanomed. 7, 835–844. 10.2147/IJN.S28278 22359460 PMC3284226

[B128] Yang X. Xu B. Zhang X. Song X. Chen R. (2014). Preparation of micro/nanostructure TiO_2_ spheres by controlling pollen as hard template and soft template. J. Nanosci. Nanotechnol. 14, 7228–7233. 10.1166/jnn.2014.8922 25924395

[B129] Yao Y. C. Zhan X. Y. Zhang J. Zou X. H. Wang Z. H. Xiong Y. C.. (2008). A specific drug targeting system based on polyhydroxyalkanoate granule binding protein PhaP fused with targeted cell ligands. Biomaterials 29, 4823–4830. 10.1016/j.biomaterials.2008.09.008 18824258

[B130] Yoshino T. Maeda Y. Matsunag T. (2010). Bioengineering of bacterial magnetic particles and their applications in biotechnology. Recent Pat. Biotechnol. 4, 214–225. 10.2174/187220810793611455 21171958

